# Correction: Functionalized pyridine in pyclen-based iron(iii) complexes: evaluation of fundamental properties

**DOI:** 10.1039/d1ra90136b

**Published:** 2021-08-13

**Authors:** Magy A. Mekhail, Kristof Pota, Timothy M. Schwartz, Kayla N. Green

**Affiliations:** Department of Chemistry and Biochemistry, Texas Christian University 2950 W. Bowie Fort Worth TX 76129 USA kayla.green@tcu.edu

## Abstract

Correction for ‘Functionalized pyridine in pyclen-based iron(iii) complexes: evaluation of fundamental properties’ by Magy A. Mekhail *et al.*, *RSC Adv.*, 2020, **10**, 31165–31170. DOI: 10.1039/d0ra05756h.

In the original article, in Table 2, incorrect values for the models for Fe(L4) were given. The correct values for the models for Fe(L4) are given in Table 2 here.

**Table:** Formation constants (log *β*) of the ML species formed, Fe(ii)/(iii)

Complex	log *β*_red_	log *β*_ox_
	Fe(ii)^a^	Fe(iii)^b^
[Fe( 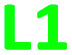 )]	14.18(5)	14.57
[Fe( 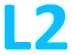 )]	14.46(7)	14.70
[Fe( 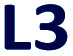 )]	12.41(2)	12.02
[Fe( 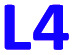 )]	13.11(2)	12.91
[Fe( 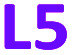 )]	12.30(2)	11.44

a *I* = 0.15 M NaCl and *T* = 25.0 °C.

b Derived from eqn (1) (ref. 31) and (Fe^2+^)_aq,_*E*°′Fe^III^/Fe^II^ = −0.439 *vs.* [Fe(CN)_6_]^3−^/[Fe(CN)_6_]^4−^.

The Royal Society of Chemistry apologises for these errors and any consequent inconvenience to authors and readers.

## Supplementary Material

